# Shared Fryers May Not Always Be as Risky for Gluten Cross-Contact in Celiac Disease as Dietary Guidance Suggests

**DOI:** 10.3390/nu18203433

**Published:** 2026-10-20

**Authors:** Vanessa Maltin Weisbrod, Meghan Donnelly McKeon, Marisa Gallant Stahl, Monique Germone, Sadie Nagle, Jackie Jossen, Jessica Lebovits, Maureen M. Leonard, Joanna Perl, Marilyn Grunzweig Geller, Aaron Geller, Luke Emerson-Mason, Zhaoxing Pan, Ritu Verma, Edwin Liu

**Affiliations:** 1 Celiac Disease Foundation, Woodland Hills, CA 91367, USAmarilyn.geller@celiac.org (M.G.G.);; 2 Children’s Hospital Colorado, University of Colorado, Aurora, CO 80045, USA; 3 Celiac Disease Center, Columbia University Medical Center, New York, NY 10032, USAjsp2212@cumc.columbia.edu (J.P.); 4 Mass General Brigham for Children, Boston, MA 02114, USA; 5 Vanderbilt Health, Nashville, TN 37232, USA; 6 Bia Diagnostics, Colchester, VT 05446, USA; 7 Celiac Disease Center, University of Chicago, Chicago, IL 60637, USA

**Keywords:** celiac disease, gluten cross-contact, shared fryers, gluten-free diet, food service practices, cumulative exposure

## Abstract

**Background:** Shared fryers are commonly perceived as a high-risk source of gluten cross-contact for individuals with celiac disease (CeD), leading to widespread avoidance of foods such as French fries and tortilla chips. However, limited real-world data exist evaluating gluten concentrations in foods prepared in shared fryers under routine food service conditions. Because long-term health outcomes in celiac disease depend on minimizing cumulative dietary gluten exposure and intestinal injury is dose-dependent, characterizing gluten contamination from common dining practices has important implications for nutritional management and disease counseling. **Objective:** The aim of this study was to quantify gluten concentrations in naturally gluten-free (GF) foods prepared in shared fryers across U.S. food service establishments and to evaluate how fryer management practices, restaurant type, and geographic distribution influence gluten content. **Methods:** This cross-sectional observational study analyzed gluten concentrations in 98 samples of French fries (*n* = 55) and tortilla chips (*n* = 43) collected from fast food, fast casual, casual dining, and fine dining establishments, cafés, and other food service settings across multiple U.S. states. All samples were naturally GF prior to frying and prepared in fryers shared with gluten-containing foods. Gluten concentrations were measured using an enzyme-linked immunosorbent assay (ELISA). Recorded variables included sample type, restaurant type, state location, and fryer oil change frequency (daily, weekly, or unknown). In accordance with the U.S. Food and Drug Administration (FDA) GF labeling standard, foods containing <20 ppm gluten were considered GF, while values ≥20 ppm were classified as exceeding the GF threshold. Values <5 ppm were considered below the assay’s limit of quantification (LOQ). **Results:** Of the 98 samples analyzed, six (6.1%) exceeded the FDA GF threshold of 20 ppm. The majority of samples (84/98, 85.7%) measured below the assay’s lower LOQ (<5 ppm gluten). All 43 tortilla chip samples tested <5 ppm. Among samples from fast food establishments, 9/12 (75.0%) measured <5 ppm, 2/12 (16.7%) measured 5–20 ppm, and 1/12 (8.3%) exceeded 20 ppm. Among samples from fast casual establishments, 13/15 (86.7%) measured <5 ppm, 1/15 (6.7%) measured 5–20 ppm, and 1/15 (6.7%) exceeded 20 ppm. Greater variability was observed in casual dining establishments, including samples exceeding 20 ppm. All samples collected from establishments reporting daily oil changes measured <20 ppm, while samples from establishments reporting weekly oil changes demonstrated higher and more variable gluten concentrations, including values greater than 20 ppm. **Conclusions:** In this multi-site observational study, shared fryers were not uniformly associated with meaningful gluten cross-contact. Gluten concentrations varied by food service context and fryer management practices, with consistently low or below-LOQ gluten levels observed in establishments reporting daily fryer oil replacement, and greater variability observed in settings with less frequent oil changes. These results indicate that shared fryers are not inherently high-risk sources of gluten cross-contact and highlight daily fryer oil replacement as a key operational factor significantly associated with lower measured gluten levels. These findings have direct nutritional implications. Estimating gluten concentrations in shared-fryer environments allows for more accurate assessment of potential daily gluten intake, which is the key determinant of mucosal healing, serologic normalization, and long-term nutritional and health outcomes in individuals with celiac disease.

## 1. Introduction

Celiac disease (CeD) is a chronic immune-mediated disorder triggered by ingestion of gluten, a protein found in wheat, barley, and rye. Strict, lifelong adherence to a gluten-free (GF) diet is currently the only effective treatment. Chronic regular ingestion of small amounts of gluten can lead to persistent enteropathy, symptoms, and long-term complications [1].

While food labeling regulations have improved access to GF packaged foods, gluten cross-contact in food service environments remains a major source of inadvertent gluten exposure. Shared kitchen equipment is frequently cited in clinical counseling as a source of risk [2,3]. Shared fryers, in particular, are often categorically discouraged due to concerns that gluten-containing batter and breading contaminate fryer oil and transfer gluten to otherwise GF foods [4].

However, empirical evidence supporting universal avoidance of shared fryers is limited. Prior small-scale studies have demonstrated variable gluten transfer depending on food matrix, cooking conditions, and study design [4,5]. Prior investigations have relied on simulated environments or limited sampling, reducing their applicability to real-world food service practices [4].

Current recommendations for adherence to the GF diet have been shaped largely by a combination of clinical experience, expert consensus, and regulatory standards designed to minimize risk in the absence of complete exposure data [5,6]. The United States (U.S.) Food and Drug Administration (FDA) GF labeling threshold of less than 20 ppm was established based on population-level modeling of cumulative gluten exposure and observed tolerance limits, rather than on discrete toxicity thresholds or systematic measurement of gluten levels across specific real-world eating scenarios [7,8]. Accordingly, the 20 ppm threshold is a regulatory standard for GF labeling and should not be interpreted as a discrete clinical safety threshold applicable to every individual with CeD. Similarly, many patient-facing dietary recommendations, particularly those addressing eating outside the home and avoidance of shared equipment, have evolved from a precautionary framework intended to protect individuals with CeD from inadvertent gluten exposure, even when empirical exposure data are limited [5,6].

As a result, guidance surrounding common food service practices, such as the use of shared fryers, has often been binary in nature, categorizing exposures as either “safe” or “unsafe” without quantitative characterization of actual gluten levels. While this approach appropriately prioritizes safety, it may not fully reflect the heterogeneity of food service operations or the range of gluten exposure magnitudes encountered in real-world settings. Overly conservative guidance may also contribute to dietary burden, anxiety, and reduced quality of life for individuals with CeD, particularly when recommendations are not grounded in measured exposure data [9,10,11].

In celiac disease, dietary gluten exposure is not merely a regulatory concern but a biologically meaningful determinant of intestinal injury. Importantly, gluten concentration expressed in parts per million does not independently define the clinical relevance of an exposure. The amount of gluten ingested depends on both the measured concentration and the quantity of food consumed, while potential biological effects must also be considered in the context of exposure frequency and cumulative dietary gluten intake.

Prospective dose–response studies demonstrate that ingestion of as little as 10 mg of gluten per day may be sufficient to induce measurable villous injury in some individuals, while higher daily doses are consistently associated with persistent enteropathy [12]. Observational studies further suggest that many patients who report strict adherence to a gluten-free diet continue to ingest measurable amounts of gluten, contributing to incomplete mucosal recovery [2,13]. Thus, quantitative assessment of gluten cross-contact should consider the magnitude and frequency of exposure rather than an isolated concentration alone.

Despite the central role of the GF diet in the management of CeD, relatively few studies have systematically quantified gluten cross-contact in everyday food preparation environments encountered by patients [14]. Empirical data describing both the frequency and magnitude of gluten exposure in shared food service settings are needed to complement existing regulatory standards and to support evidence-informed counseling that balances safety with feasibility. This gap is especially relevant for scenarios such as shared fryers, which are widely encountered, operationally variable, and frequently cited by patients as a source of uncertainty when dining outside the home.

To address this gap, this study was designed to quantify gluten concentrations in naturally GF foods (French fries and tortilla chips) prepared in shared fryers under routine operating conditions across diverse U.S. food service establishments. Rather than treating shared fryers as a binary risk factor, this study evaluates how restaurant type and fryer management practices influence gluten cross-contact risk within the framework of existing FDA GF standards.

## 2. Materials and Methods

This cross-sectional observational study was conducted according to a prespecified protocol developed by the Celiac Disease Foundation and co-investigators, including staff and healthcare providers from pediatric and adult CeD centers, staff from the Celiac Disease Foundation, and registered dietitians from the Dietitian Advisory Council of the Society for the Study of Celiac Disease. The study did not involve human subjects and was exempt from institutional review board approval.

Samples were collected between September 2025 and January 2026 from food service establishments located in 14 U.S. states: California, Colorado, Georgia, Kansas, Massachusetts, Michigan, Missouri, New Jersey, New Mexico, New York, Pennsylvania, Tennessee, Virginia, and Washington. Eligible samples met all of the following criteria:

- The food item was naturally GF prior to frying (French fries or tortilla chips);
- The fryer was used to prepare both gluten-containing and GF foods;
- The fryer was not designated as GF.

Collectors were instructed to use clean hands prior to handling the samples to prevent any inadvertent transfer of gluten. For each sample, sufficient food was collected to fill a sandwich-sized seal-top bag (16.5 cm × 14.9 cm). Samples were shipped to Bia Diagnostics in Colchester, Vermont, for analysis. Each of the collected food samples was submitted to the laboratory and processed independently. Each sample was homogenized separately in its entirety prior to analysis, and a 1 g analytical test portion was obtained from the resulting homogenate. Samples were not pooled or combined.

Gluten concentration was measured at Bia Diagnostics, an ISO 17025-accredited laboratory [15], using the R-Biopharm RIDASCREEN^®^ Gliadin ELISA (R7001; R-Biopharm AG, Darmstadt, Germany) according to the AOAC International Official Method of Analysis 2012.01. The assay uses the R5 monoclonal antibody with Mendez Cocktail extraction, which together comprise the Codex Type I method for gluten quantification [16]. The assay is calibrated to Prolamin Working Group gliadin, with gluten concentration calculated using the Codex conversion of 2 × [gliadin] = [gluten]. The quantitative range of the assay is 5–80 ppm gluten, with a limit of quantification (LOQ) of 5 ppm. Results below the LOQ are reported as <5 ppm, while results above the upper quantitative limit are reported as >80 ppm. Positive and negative controls were included with each analytical batch and results were monitored over time for trends as part of the laboratory’s quality-control procedures. The AOAC collaborative validation included corn-based products, starches, and potatoes, and the method has additionally been validated in-house by Bia Diagnostics for fried-food matrices.

At the time of sample collection, the collector asked food service staff whether the sampled food item was naturally GF prior to frying, whether it was prepared in a shared fryer with gluten-containing foods, and about fryer oil replacement frequency. Information was obtained through direct communication with establishment staff. All collectors acknowledged that these questions were asked at the time of sampling and recorded responses using a standardized collection form.

Restaurant names and staff identifiers were anonymized prior to analysis.

Food samples were collected from a range of food service establishments and categorized by restaurant type using standard industry descriptors and self-reported characteristics at the time of sampling. Categories were defined as follows:

- Fine Dining: Full-service restaurants with table service and higher price points.
- Casual Dining (not a chain): Independently owned, full-service restaurants without franchised branding.
- Fast Casual: Limited-service restaurants offering customizable menu options.
- Fast Food: Limited-service restaurants with standardized menus and high customer throughput.
- Café: Establishments primarily focused on beverages with a limited food menu.
- Chain Restaurant/Franchise: Branded restaurants operating across multiple locations with standardized menus and procedures.
- Other: Establishments not fitting into the above categories or representing hybrid service models.

Gluten concentrations were evaluated descriptively by restaurant type, fryer oil change frequency, sample type, and geographic location. Concentrations were grouped as <5 ppm, 5 to <20 ppm, and ≥20 ppm based on the assay LOQ and FDA GF labeling threshold. Given the sample size and sparse observations within several strata, formal hypothesis testing was limited to selected comparisons. Associations between sample characteristics and gluten concentrations ≥20 ppm were evaluated using Fisher’s exact test due to small cell counts.

Finally, gluten concentrations were interpreted within the framework of established dose–response data linking cumulative daily gluten intake to mucosal outcomes in celiac disease, allowing measured ppm values to be contextualized in terms of potential dietary exposure.

## 3. Results

A total of 98 naturally GF food samples were collected from shared-fryer environments across multiple U.S. states. Samples included 55 French fry samples (56.1%) and 43 tortilla chip samples (43.9%). All foods were naturally GF prior to frying and prepared in fryers shared with gluten-containing foods.

Samples were collected from a range of food service establishment types, including casual dining restaurants (*n* = 44), chain restaurants/franchises (*n* = 19), fast casual establishments (*n* = 15), and fast food establishments (*n* = 12), as well as a smaller number of fine dining restaurants (*n* = 2), cafés (*n* = 2), and other food service settings (*n* = 4).

Fryer oil replacement frequency was reported to be daily for 39 samples (39.8%), weekly for 35 samples (35.7%), and unknown for 24 samples (24.5%).

Across all samples prepared in shared fryers (*n* = 98), six samples (6.1%) exceeded the FDA GF threshold of 20 ppm, with measured concentrations of 29.6 ppm, 34.9 ppm, 67.9 ppm, and >80 ppm in three samples. The remaining 92 samples (93.9%) contained <20 ppm gluten. Eight samples (8.2%) measured between 5 and <20 ppm gluten, falling below the FDA GF threshold but above the assay’s lower LOQ. Using an exact binomial model, the estimated probability of a greater than 20 ppm result in a shared fryer was 6.1% (95% confidence interval: 2.3–12.9%). Notably, 84 samples (85.7%) measured below the assay’s lower LOQ (Table 1).

When stratified by food type, all samples exceeding the FDA GF threshold were French fries (10.9%), whereas no tortilla chip samples exceeded 20 ppm. This difference was statistically significant (Fisher’s exact test, *p* = 0.033).

Among samples with known fryer oil replacement frequency, none of the foods collected from establishments reporting daily oil changes exceeded the FDA GF threshold of 20 ppm, whereas four samples collected from establishments reporting weekly oil changes exceeded 20 ppm (11.4%) (Table 2). The difference between daily and weekly oil replacement frequencies was statistically significant according to Fisher’s exact test (*p* = 0.046).

Gluten concentrations were distributed across multiple restaurant types, with the majority of samples in all categories except for fine dining measuring below 5 ppm and no clear clustering of samples exceeding 20 ppm by establishment type (Table 3).

Samples were collected from restaurants located in 14 U.S. states. Samples exceeding 20 ppm were observed in more than one state, with no consistent geographic clustering.

## 4. Discussion

In this multi-site observational study, gluten cross-contact in shared fryers was uncommon but not absent. Across 98 samples of naturally GF foods prepared in shared fryers, the majority (93.9%) contained less than 20 ppm gluten, meeting the FDA GF labeling standard. The overall probability of a sample exceeding 20 ppm was approximately 6.1%, indicating that shared fryers are not uniformly high-risk sources of gluten cross-contact, but rather represent a context-dependent exposure that varies by food service practice. When the analysis was restricted to fries, 11% (6/55) of samples exceeded 20 ppm. Overall, these findings are consistent with previous studies demonstrating that gluten transfer during shared food preparation is variable rather than uniform across foods and preparation conditions [4,5,6]. An additional observation of note was that all tortilla chip samples measured below the assay’s lower LOQ of <5 ppm gluten.

Importantly, fryer oil replacement frequency emerged as a significant operational factor associated with gluten cross-contact risk. All samples exceeding the FDA GF threshold were collected from establishments reporting weekly oil changes, whereas no exceedances were observed in establishments reporting daily oil replacement. This association reached statistical significance and suggested that more frequent oil replacement may limit cumulative gluten accumulation in shared-fryer environments. While causality cannot be inferred from this observational design, the consistency of this pattern supports fryer oil management as a meaningful contributor to observed variability in gluten exposure.

Beyond the proportion of samples exceeding 20 ppm, the magnitude of measured gluten concentrations provides additional context for interpreting potential exposure. Among the six samples exceeding 20 ppm, concentrations ranged from 29.6 ppm to >80 ppm, with three samples exceeding the assay’s upper reporting limit of 80 ppm. These findings demonstrate that although concentrations ≥20 ppm were uncommon in the overall sample, the magnitude of gluten cross-contact varied considerably when it did occur. Interpretation of these concentrations must also consider the quantity of food consumed, exposure frequency, and cumulative dietary gluten intake.

The FDA GF labeling standard permits foods containing <20 ppm gluten. However, this regulatory threshold should not be interpreted as establishing clinical safety for an individual patient. The potential clinical relevance of an exposure depends not only on gluten concentration, but also on the quantity consumed, exposure frequency, cumulative dietary gluten intake, and individual variability.

In celiac disease, the clinical relevance of measured gluten concentrations depends on total daily intake and exposure frequency rather than isolated ppm values. Controlled gluten challenge studies suggest that sustained ingestion of approximately 10 mg of gluten per day may result in histologic changes in susceptible individuals, whereas lower or intermittent exposures may not produce measurable mucosal injury [12]. Translating measured ppm concentrations into estimated intake per portion provides clinically meaningful context.

However, gluten exposure in real-world settings is cumulative. Repeated daily intake from multiple low-level sources, including packaged foods near the regulatory threshold, cross-contact in shared kitchens, and restaurant meals, may collectively contribute to persistent enteropathy in a subset of patients. Studies using stool and urine gluten immunogenic peptide detection have demonstrated that measurable gluten ingestion occurs frequently even among patients who consider themselves highly adherent to the gluten-free diet [13].

Accordingly, quantifying the magnitude and frequency of gluten exposure from common food service practices is directly relevant to nutritional counseling, dietary adequacy, and long-term health outcomes. Rather than framing shared fryer exposure as categorically safe or unsafe, interpreting measured gluten levels within a dose–response framework allows clinicians and dietitians to better estimate potential contributions to total dietary gluten intake.

As a practical illustration, consumption of approximately 500 g of food containing gluten at 20 ppm would theoretically correspond to approximately 10 mg of gluten [12]. This calculation assumes homogeneous gluten distribution throughout the food and consumption of the entire 500 g portion at the measured concentration. It is intended to illustrate the relationship between measured concentration, quantity consumed, and estimated gluten dose rather than to define an individual clinical safety threshold.

Accordingly, consumption of multiple foods in a single day that meet the GF standard, even when individual products contain gluten levels near the upper limit, would generally be expected to remain within ranges considered acceptable by regulators and clinicians, assuming customary portion sizes. For example, an occasional meal prepared in a shared fryer that yields gluten levels near or modestly above 20 ppm, particularly when consumed infrequently, is unlikely to represent a major contributor to cumulative gluten exposure compared with more common sources of inadvertent daily exposure (i.e., shared water for cooking pasta, shared condiments, and dirty kitchen equipment). This type of intake reflects the normal way people eat across a day and does not indicate that an individual has done anything wrong or placed themselves at immediate risk, particularly when such exposures are occasional rather than repeated daily over long periods of time [12].

Effective nutritional management of celiac disease must balance strict gluten avoidance with dietary sustainability. Excessively restrictive counseling may reduce dietary variety, impair nutritional adequacy, and increase psychosocial burden, potentially undermining long-term adherence. Difficulty eating outside the home has consistently been associated with reduced quality of life and increased dietary anxiety [9,10,11]. Because adherence is a major determinant of long-term mucosal healing, counseling approaches that appropriately stratify exposure risk may support both nutritional sufficiency and sustained health outcomes.

Thus, providers should consider a counseling framework that differentiates between frequent, routine sources of gluten exposure and occasional, low-level exposures. When foods prepared in shared fryers demonstrate <5 ppm gluten, and when consumption is infrequent, such exposures might be considered low-risk and are unlikely to represent a major source of cumulative gluten intake. It is well documented that symptoms are not a reliable indicator of mucosal activity, so counseling that incorporates exposure frequency, cumulative dose, and food service practices could allow patients to make informed decisions that balance medical risk with psychosocial well-being [13].

Importantly, individual physician- or dietitian-led counseling approaches that acknowledge variability in risk may help reduce unnecessary dietary restriction, improve confidence in eating outside the home, and support long-term adherence to the GF diet. This approach aligns with principles of shared decision-making and recognizes that individual tolerance for risk varies, while still prioritizing avoidance of frequent or repeated gluten exposure, which is known to drive disease activity.

A notable strength of this study is the analytical sample size used for gluten detection. Each food sample was analyzed using a 1 g test portion, substantially larger than the approximately 0.25 g sample sizes commonly used in routine food manufacturing gluten testing and thereby reducing potential sampling errors. Fried foods may exhibit heterogeneous or localized gluten contamination, and smaller test portions may fail to capture sporadic contamination. The use of a larger analytical sample reduces the likelihood of false-negative results and increases confidence that low or below-LOQ measurements reflect true absence of meaningful gluten accumulation.

This study has several limitations that should be considered when interpreting the findings. Participating establishments were not randomly selected, introducing the possibility of selection bias and limiting generalizability to all food-service settings. Oil replacement frequency and other fryer practices were reported by restaurant personnel and were not independently verified. Each sample was obtained from a different establishment, and establishments were sampled only once; therefore, the study does not assess within-establishment or within-fryer variability over time or under different operating conditions. Finally, only six samples exceeded 20 ppm, limiting the ability to draw conclusions about factors associated with higher gluten concentrations. However, this approach reflects the information typically available to consumers and clinicians when making dietary decisions. Other operational characteristics, including fryer design, the amount and type of gluten-containing foods prepared in the fryer, oil volume, filtration practices, food turnover, temperature, and cleaning procedures, were not assessed in this study. Whether and to what extent these factors influence gluten cross-contact in shared fryers is unknown and warrants further investigation.

Although very high-temperature food processing can alter gluten protein structure and, in some contexts, affect ELISA detectability, particularly in extensively hydrolyzed or fermented products such as beer, soy sauce, or malt-based ingredients, the temperatures used in commercial fryers are not known to reliably impair detection using standard sandwich ELISA methods. Competitive ELISA methods designed for hydrolyzed gluten have been proposed in some settings [4]. However, these assays are not currently recognized by the FDA as appropriate for regulatory GF determination and have been reported to yield higher and potentially exaggerated gluten values in certain food matrices [17,18]. Differences in sampling strategy, food matrices, study design, and testing conditions limit direct comparison with previous shared-fryer studies, including Thompson et al. [4]. Accordingly, use of the standard sandwich ELISA in this study aligns with current FDA regulatory guidance and is appropriate for assessing clinically and regulatory relevant gluten exposure in fried-food matrices [8,19]. Importantly, the study was not designed to assess clinical outcomes, symptom provocation, or mucosal response, and the findings should not be interpreted as evidence of individual tolerance or safety [20].

Although samples were collected across multiple states and restaurant types, the study does not encompass all possible food service settings or preparation practices. Larger studies with repeated sampling and integration of clinical outcome data may help to further refine risk estimates and better characterize the relationship between measured gluten exposure and clinical significance.

Finally, future studies integrating repeated dietary exposure measurement with serologic and histologic outcomes would further clarify the relationship between quantified environmental gluten exposure and long-term nutritional health.

## 5. Conclusions

This study provides quantitative data on gluten concentrations in foods prepared in shared-fryer environments and places these findings within a nutritional exposure framework relevant to health outcomes in celiac disease. Because mucosal healing and long-term complication risk are driven by cumulative daily gluten intake, understanding the magnitude and frequency of real-world dietary exposures is essential. The present findings suggest that shared-fryer exposure is context-dependent and may contribute variably to total dietary gluten intake depending on operational practices.

Importantly, while this study was not designed to assess clinical outcomes or long-term health effects, the results offer evidence to support nutritional counseling conversations focused on dietary adherence, particularly discussions that distinguish between frequent, routine sources of gluten exposure and lower-probability, context-dependent exposure encountered in certain food service settings. Applying this evidence-informed perspective in CeD care may help clinicians and dietitians address patient concerns, reduce unnecessary dietary burden, and support long-term adherence to the GF diet.

## Figures and Tables

**Table 1:** Distribution of Gluten Concentrations in Foods Prepared in Shared Fryers.

Gluten Concentration	Number of Samples, *n* (%)
<5 ppm	84 (85.7%)
5–20 ppm	8 (8.2%)
≥20 ppm	6 (6.1%)
Total	98 (100%)

**Table 2:** Gluten concentrations by fryer oil change frequency.

Oil Change Frequency	<5 ppm, *n* (%)	5–20 ppm, *n* (%)	≥20 ppm, *n* (%)
Daily (*n* = 39)	37 (82.2%)	2 (5.1%)	0 (0.0%)
Weekly (*n* = 35)	26 (74.3%)	5 (14.3%)	4 (11.4%)
Unknown (*n* = 24)	21 (87.5%)	1 (4.2%)	2 (8.3%)
Total (*n* = 98)	84 (85.7%)	8 (8.2%)	6 (6.1%)

**Table 3:** Gluten concentrations by restaurant type.

Restaurant Type	<5 ppm, *n* (%)	5–20 ppm, *n* (%)	≥20 ppm, *n* (%)
Casual dining (not a chain) (44)	36 (81.8%)	5 (11.4%)	3 (6.8%)
Chain restaurant/franchise (19)	19 (100%)	0 (0.0%)	0 (0.0%)
Fast casual (15)	13 (86.7%)	1 (6.7%)	1 (6.7%)
Fast food (12)	9 (75.0%)	2 (16.7%)	1 (8.3%)
Fine dining (2)	1 (50.0%)	0 (0.0%)	1 (50.0%)
Café (2)	2 (100%)	0 (0.0%)	0 (0.0%)
Other (4)	4 (100%)	0 (0.0%)	0 (0.0%)
Total (98)	84 (85.7%)	8 (8.2%)	6 (6.1%)

## Data Availability

The raw data supporting the conclusions of this article will be made available by the authors upon request.

## Abbreviations

The following abbreviations are used in this manuscript:

CeD	celiac disease
GF	gluten-free
FDA	U.S. Food and Drug Administration
U.S.	United States
ELISA	enzyme-linked immunosorbent assay
ppm	parts per million
